# MEndoB, a chimeric lysin featuring a novel domain architecture and superior activity for the treatment of staphylococcal infections

**DOI:** 10.1128/mbio.02540-23

**Published:** 2024-01-26

**Authors:** Christian Roehrig, Markus Huemer, Dominique Lorgé, Fabienne Arn, Nadine Heinrich, Lavanja Selvakumar, Lynn Gasser, Patrick Hauswirth, Chun-Chi Chang, Tiziano A. Schweizer, Fritz Eichenseher, Steffi Lehmann, Annelies S. Zinkernagel, Mathias Schmelcher

**Affiliations:** 1 Micreos Pharmaceuticals AG, Baar, Zug, Switzerland; 2 Micreos GmbH, Wädenswil, Zurich, Switzerland; 3 Institute of Chemistry and Biotechnology, Zurich University of Applied Sciences (ZHAW), Wädenswil, Zurich, Switzerland; 4 Department of Infectious Diseases and Hospital Epidemiology, University Hospital Zurich, University of Zurich, Zurich, Switzerland; St Jude Children's Research Hospital, Memphis, Tennessee, USA

**Keywords:** antibiotic resistance, endolysin, bacteriophage, MRSA, bacteremia, peptidoglycan hydrolase, protein therapeutics, antibiotics, staphylococci, *Staphylococcus aureus*

## Abstract

Bacterial infections are a growing global healthcare concern, as an estimated annual 4.95 million deaths are associated with antimicrobial resistance (AMR). Methicillin-resistant *Staphylococcus aureus* is one of the deadliest pathogens and a high-priority pathogen according to the World Health Organization. Peptidoglycan hydrolases (PGHs) of phage origin have been postulated as a new class of antimicrobials for the treatment of bacterial infections, with a novel mechanism of action and no known resistances. The modular architecture of PGHs permits the creation of chimeric PGH libraries. In this study, the chimeric enzyme MEndoB was selected from a library of staphylococcal PGHs based on its rapid and sustained activity against staphylococci in human serum. The benefit of the presented screening approach was illustrated by the superiority of MEndoB in a head-to-head comparison with other PGHs intended for use against staphylococcal bacteremia. MEndoB displayed synergy with antibiotics and rapid killing in human whole blood with complete inhibition of re-growth over 24 h at low doses. Successful treatment of *S. aureus*-infected zebrafish larvae with MEndoB provided evidence for its *in vivo* effectiveness. This was further confirmed in a lethal systemic mouse infection model in which MEndoB significantly reduced *S. aureus* loads and tumor necrosis factor alpha levels in blood in a dose-dependent manner, which led to increased survival of the animals. Thus, the thorough lead candidate selection of MEndoB resulted in an outstanding second-generation PGH with *in vitro*, *ex vivo,* and *in vivo* results supporting further development.

**IMPORTANCE:**

One of the most pressing challenges of our era is the rising occurrence of bacteria that are resistant to antibiotics. Staphylococci are prominent pathogens in humans, which have developed multiple strategies to evade the effects of antibiotics. Infections caused by these bacteria have resulted in a high burden on the health care system and a significant loss of lives. In this study, we have successfully engineered lytic enzymes that exhibit an extraordinary ability to eradicate staphylococci. Our findings substantiate the importance of meticulous lead candidate selection to identify therapeutically promising peptidoglycan hydrolases with unprecedented activity. Hence, they offer a promising new avenue for treating staphylococcal infections.

## INTRODUCTION

*Staphylococcus aureus* is an opportunistic human pathogen that colonizes approximately 30% of individuals permanently in the nares (1–3). Besides being the main cause of skin and soft tissue infections (SSTIs), *S. aureus* can cause severe and life-threatening infections, such as bacteremia and endocarditis (1, 4–6). Although local skin infections are most of the time self-limiting, SSTIs are the most commonly identified cause of *S. aureus* bloodstream infections (SBIs) (6, 7). In addition, *S. aureus* can easily develop resistance to antibiotics as well as evade killing by conventional antibiotics by forming biofilms (8), abscesses (9), hiding inside eukaryotic cells (10), and forming antibiotic-tolerant persister cells (11–14). Infections with methicillin-resistant *S. aureus* (MRSA) that result in hospitalization are the cause of over 18,000 annual deaths in the USA alone (15, 16), and by developing resistance to antibiotics (16–19), effective treatment becomes even more difficult.

While *S. aureus* is one of the top three leading causes of sepsis and sepsis-related deaths (5, 20), coagulase-negative staphylococci, especially *Staphylococcus epidermidis*, are also found as a frequent cause of bacteremia, specifically in neonates (5, 21, 22). *S. epidermidis* is a strong biofilm former and thus one of the most frequent causes of medical implant-associated infections (IAIs), including orthopedic implants, pacemakers, prosthetic heart valves, and vascular grafts (23). These infected devices can be the source of bacterial dissemination and bacteremia (5, 24, 25).

Despite treatment with antibiotics, SBIs still result in high mortality rates of 10%–30%, frequent relapses (5%–10%), and lasting impairments in more than a third of the survivors (3, 16, 26, 27). Hematogenous spread of staphylococci may also lead to additional complications such as abscesses, osteomyelitis, spondylodiscitis, endocarditis, and IAIs, which can occur weeks or months after the primary infection. Patients suffering from prolonged bacteremia episodes have an increased risk of secondary infection foci (28). Additionally, inadequate antibiotic therapy, unknown primary infection foci, and insufficient source control can further increase the risk for complications (13, 25, 29).

Peptidoglycan hydrolases (PGHs) cleave specific bonds within the peptidoglycan (PG) network of bacteria and have been shown to be active against biofilms (30). Their high lytic activity makes PGHs potent anti-staphylococcal agents. Endolysins are phage-derived, highly specific PGHs, active against both drug-susceptible and resistant bacteria (31). Systematic engineering and screening approaches to improve and select PGHs for specific applications have previously been described (32). As potential alternatives to antibiotics, they have undergone *in vitro*, *in vivo,* and investigations in clinical studies, where they reliably displayed a benign safety profile (33).

**Fig 1:**
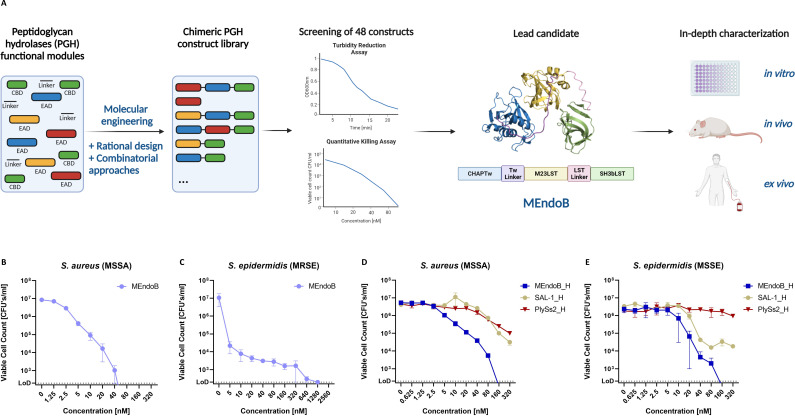
Selection of MEndoB from a library of chimeric staphylococcal PGHs. A chimeric PGH library of 48 constructs was cloned, expressed, and characterized (**A**). Constructs were screened for activity in PBS and human serum with two orthogonal assays (turbidity reduction assay and quantitative killing assay [qKA]) against *S. aureus* and *S. epidermidis*. The most active construct (MEndoB) was further tested *in vitro*, *ex vivo,* and *in vivo*. MEndoB comprises two EADs (N-terminal CHAP domain [blue] and the central M23 endopeptidase domain [yellow]), one CBD (C-terminal SH3b domain [green]), and two linkers (purple and pink) from different origins, shown in a 3D-modeled ribbon representation (ColabFold). Activity of MEndoB against *S. aureus* ATCC 12600 (**B**) and *S. epidermidis* ATCC 35984 (**C**) in human serum, as determined by qKAs. Activity of C-terminally His-tagged versions of MEndoB, SAL-1, and PlySs2 in human serum against *S. aureus* ATCC 12600 (**D**) and *S. epidermidis* ATCC 12228 (**E**), determined by qKAs. The limit of detection was 200 CFUs/mL (dotted line), and error bars represent standard error of the mean from four (**B and C**) and two (**D and E**) biological replicates. Figure was modified from reference 32 and created using biorender.com.

PGHs of staphylococci display a modular architecture, consisting of enzymatically active domains (EADs) and cell-wall-binding domains (CBDs). The high specificity of staphylococcal PGHs may, in part, be attributed to their CBDs, which regularly feature a SH3b-like protein domain structure. The structures of staphylococcal endolysin CBDs have been solved and display great homology to the SH3b-like CBDs of the bacteriocin lysostaphin (LST) and ALE1, suggesting a common recognition site in the PG (34, 35). The EADs are more diverse and can be grouped according to their cleavage sites within the PG. In this study, we investigated chimeric PGHs consisting of two different EAD types, the cysteine, histidine-dependent amidohydrolase/peptidase (CHAP) and the M23 endopeptidase domain. CHAP domains are frequently found in staphylolytic endolysins, for example, in phage K or Twort (36). For nearly all CHAP domains in staphylococcal endolysins, cleavage of the PG occurs at the peptide bond between the stem peptide and the interpeptide cross bridge (37) and very rarely also at the amide bond between the sugar backbone and the stem peptide (38) (Fig. 1). Thus far, only one endolysin with an M23 endopeptidase EAD has been identified in phage 2638A; however, both staphylococcal bacteriocins, LST and its homolog ALE1, carry this domain (39). The M23 domains of LST and ALE1 cleave within the pentaglycine cross bridge, which is unique to *S. aureus* and connects adjacent stem peptides in the PG, whereas the M23 domain of phage 2638A cuts the bond between the interpeptide cross bridge and the stem peptide, which is present in the PG of all staphylococci and identical to the CHAP cleavage site of other staphylococcal endolysins (37, 40).

Previously, synergy has been observed for combinations of PGHs against *S. aureus* with orthogonal cut sites in the PG (41). A possible explanation is that the cleavage of one bond within the PG network results in better accessibility of the other and vice versa within the three-dimensional PG network. In addition, evidence has been published demonstrating a reduction in the risk for resistance development for PGHs featuring combinations of multiple EADs, including M23 and CHAP domains (42).

This study focused on the identification of MEndoB, a new chimeric anti-staphylococcal PGH featuring a novel domain architecture, and intended for systemic use and its characterization *in vitro, ex vivo,* and *in vivo*. We emphasize the importance of screening for activity under relevant physiological conditions (human serum) when selecting PGHs for later application (41, 43, 44) and provide evidence for the superiority of MEndoB compared to other non-engineered lysins.

## RESULTS

### Identification of the highly active PGH MEndoB from a chimeric PGH library by screening for activity in human serum

MEndoB was selected from a chimeric PGH library based on its superior ability to kill staphylococci in human serum. The library contained EADs and CBDs of highly active PGHs following a novel domain architecture (CHAP_M23_SH3b and M23_CHAP_SH3b), which had been identified as promising in a previous study (44). Following protein expression and purification, the activity of PGHs in the library was compared using turbidity reduction assay (TRA) and quantitative killing assays (qKA) against *S. aureus* and *S. epidermidis* (Fig. 1A).

MEndoB was readily purified by one-step CIEX (Fig. S2) and showed high, dose-dependent activity against both staphylococcal species at very low concentrations (10 nM = 0.46 µg/mL) (Fig. 1B and C). Prediction of the three-dimensional protein structure of MEndoB using ColabFold suggested that the chimeric protein assumes a highly organized structure with three functional domains separated by two flexible linkers (Fig. 1A). We compared the activity of MEndoB to other staphylococcal PGHs intended for systemic use. To this end, MEndoB and the published, non-engineered PGHs PlySs2 and SAL-1 were purified with a C-terminal His-tag and qKAs were performed in human serum (45, 46). Superior activity of MEndoB was observed against both *S. aureus* and *S. epidermidis* as MEndoB consistently killed more than one log-unit more staphylococci than the other PGHs at identical concentrations (Fig. 1D and E). These findings demonstrate that the systematic and stringent screening procedure applied here resulted in the selection of a chimeric enzyme, MEndoB, which is highly active against staphylococci in human serum and therefore holds promise for systemic application.

### MEndoB demonstrates activity against clinical staphylococcal isolates and synergy with conventionally used antibiotics

To further evaluate the potential of MEndoB for the treatment of SBIs, we determined its minimum inhibitory concentration (MIC) for a collection of 21 clinical staphylococcal isolates and reference strains and tested for synergy with antibiotics used in clinics. MIC values for six different staphylococcal species ranged from 0.008 to 0.5 µg/mL, and no significant difference in MICs was observed (*P* ≥ 0.91) when comparing methicillin-resistant *S. aureus* (MRSA) and methicillin-resistant *S. epidermidis* (MRSE) to methicillin-susceptible strains (methicillin-susceptible *S. aureus* [MSSA] and methicillin-susceptible *S. epidermidis* [MSSE]) (Table 1; Fig. S3). To determine the synergy of MEndoB with different classes of antibiotics used in clinics, we performed checkerboard assays, a two-dimensional version of the MIC, overlaying two gradients of different antimicrobials. We observed synergy with the antibiotics oxacillin, flucloxacillin, and cefazolin (fractional inhibitory concentration index, FICI ≤ 0.5) and additive effects for vancomycin, levofloxacin, and daptomycin (FICI = 0.5–1.0) (Table 1). No antagonistic effects were identified.

**TABLE 1.** Minimum inhibitory concentrations of staphylococcal strains and synergistic and additive effects of MEndoB with antibiotics^*a*^

Species	Strain origins	Number of strains (*n*)	Range of MICs (μg/mL)
*S. aureus* (MSSA)	Reference and clinical isolate strains	9	0.016–0.5
*S. aureus* (MRSA)	Reference and clinical isolate strains	4	0.016–0.25
*S. epidermidis* (MSSE)	Reference strain	1	0.031
*S. epidermidis* (MRSE)	Reference strain	1	0.031
*S. epidermidis*	Clinical isolate strain	1	0.031
*Staphylococcus pseudintermedius*	Clinical isolate strains	2	0.008–0.063
*Staphylococcus argentus*	Clinical isolate strain	1	0.125–0.5
*Staphylococcus warneri*	Clinical isolate strain	1	0.063–0.125
*Staphylococcus hominis*	Clinical isolate strain	1	0.031–0.063
			
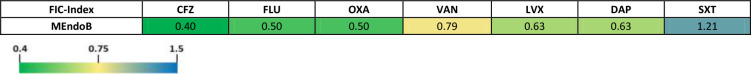			

^*a*^ The MIC range of MEndoB against clinical isolates and reference staphylococcal strains (21 strains in total) was determined. MICs were assessed in CAMHB-HSD following the CLSI guidelines. At least three biological replicates were performed. The diagram shows the fractional inhibitory concentration index of MEndoB and seven conventionally used antibiotics. Cefazolin (CFZ), flucloxacillin (FLU), oxacillin (OXA), vancomycin (VAN), levofloxacin (LVX), daptomycin (DAP), and trimethoprim/sulfamethoxazole (SXT) were tested for synergistic (FIC index ≤ 0.5), additive, or indifferent (FIC index > 0.5–4) and antagonistic (FIC index > 4) effects with MEndoB. FICI means of three biological replicates are shown.

### MEndoB shows high activity in animal sera and at physiologically relevant temperatures

**Fig 2:**
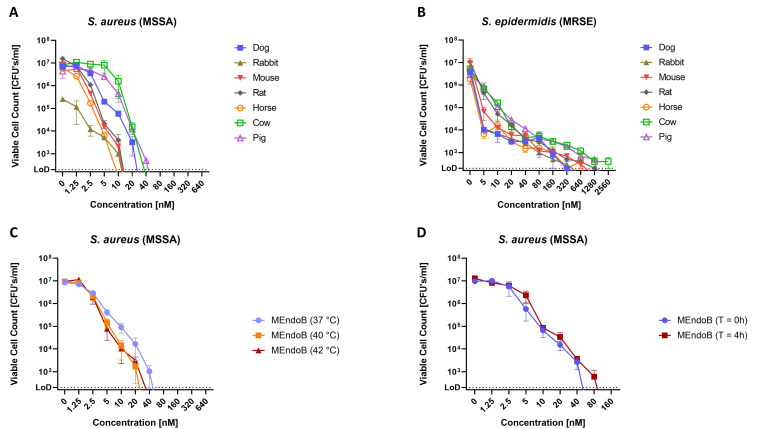
MEndoB kills different staphylococcal species in various animal sera, at elevated temperatures, and remains stable upon storage for 4 h at 37°C in human serum. MEndoB activity in qKAs at 37°C against *S. aureus* ATCC 12600 (**A**) and *S. epidermidis* ATCC 35984 (**B**) in seven different animal sera and at 37°C, 40°C, and 42°C against *S. aureus* ATCC 12600 in human serum (**C**). Data shown in panel **C** for 37°C corresponds to data from Fig. 1B. Activity of MEndoB after incubation in human serum for 4 h at 37°C was determined in qKAs at 37°C against *S. aureus* ATCC 12600 (**D**). The limit of detection was 200 CFUs/mL (dotted line), and error bars indicate the standard error of the mean from two (**A and B**) and three (**C and D**) biological replicates.

Killing assays were performed against *S. aureus* and *S. epidermidis* in seven different animal sera to evaluate their potential impact on the enzymatic activity of MEndoB and to aid the selection of suitable animal models for further preclinical development. Depending on the animal serum used, differences in activity spectra have previously been described for other PGHs (47). We generally observed high activity spectra with 10^7^ *S. aureus* colony-forming units (CFUs) reduced below detectable counts at approximately 10–40 nM throughout all sera tested (Fig. 2A). Variations between animal sera were generally small, while for *S. epidermidis*, higher concentrations were necessary to reach the detection limit (Fig. 2B). Overall, the killing patterns in human serum matched those observed in the animal sera (Fig. 1B and C), indicating the absence of a pronounced inhibitory effect of sera on MEndoB enzyme activity and suitability of multiple animals to model MEndoB activity in humans.

Elevated temperatures can be found in severely ill patients. We, therefore, tested the activity of MEndoB at 40°C and 42°C in addition to 37°C. While not statistically significant, a trend toward higher activity was observed at elevated temperatures as indicated by a shift of the activity curves to a lower concentration range (Fig. 2C). In addition, we assessed the in-use stability of MEndoB by incubating the protein in human serum at 37°C for 4 h prior to activity measurement (Fig. 2D). No significant difference was observed, demonstrating a high in-use stability profile. Overall, these results underline the potential of MEndoB for the treatment of SBI, qualifying it for further preclinical analysis.

### MEndoB eliminates *S. aureus* and *S. epidermidis* in human whole blood

**Fig 3:**
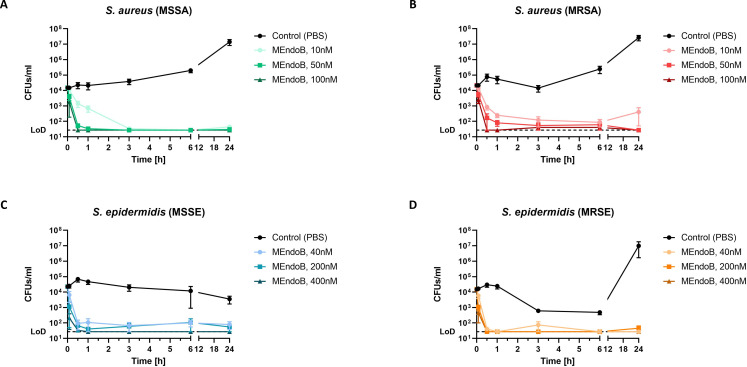
MEndoB effectively kills staphylococcal species in human whole blood. Time kill curves of different *S. aureus* and *S. epidermidis* strains in fresh human whole blood are shown. Human whole blood was inoculated with 10^4^ CFUs/mL *S. aureus* ATCC 12600 (MSSA) (**A**), *S. aureus* USA300 JE2 (MRSA) (**B**), *S. epidermidis* 1457 (MSSE) (**C**), or *S. epidermidis* ATCC 35984 (MRSE) (**D**) and MEndoB or PBS was added at three different concentrations [10, 50, and 100 nM for (**A and B**) and 40, 200, and 400 nM for (**C and D**)]. Bacterial survival was evaluated over time (5 min, 30 min, 1 h, 3 h, 6 h, and 24 h) (**A–D**). The limit of detection was 27 CFUs/mL (dotted line), and error bars show the standard error of the mean from four healthy donors. 100 nM = 4.6 µg/mL.

We inoculated freshly drawn blood from healthy volunteers with MSSA, MRSA, MSSE, and MRSE *ex vivo* to test the activity of MEndoB in human whole blood (Fig. S4). Samples were treated with three different concentrations of MEndoB, and CFU counts in the samples were monitored over time to assess a dose-dependent killing and an inhibition of the re-growth of the bacteria (Fig. 3). Rapid killing of staphylococci was observed for all concentrations after 5–30 min, illustrating the unique mode of action of PGHs as direct lytic agents in comparison to conventional antibiotics. The highest concentrations of MEndoB (100 nM for *S. aureus* and 400 nM for *S. epidermidis*) led to a sustained inhibition over 24 h for all strains tested, while PBS-treated controls remained at high levels of up to 10^7^ CFUs/mL. Even at the lowest concentrations (10 nM for *S. aureus* and 40 nM for *S. epidermidis*), a reduction below the initial inoculum was observed for all time points. No significant differences in killing by MEndoB were observed between MSSA and MRSA (Fig. 3A and B) and MSSE and MRSE (Fig. 3C and D) at matching concentrations and time points (*P* ≥ 0.16 in all cases). In conclusion, the high, dose-dependent activity of MEndoB observed in human serum was also retained in whole human blood.

### MEndoB effectively reduces *S. aureus* infections in zebrafish larvae

**Fig 4:**
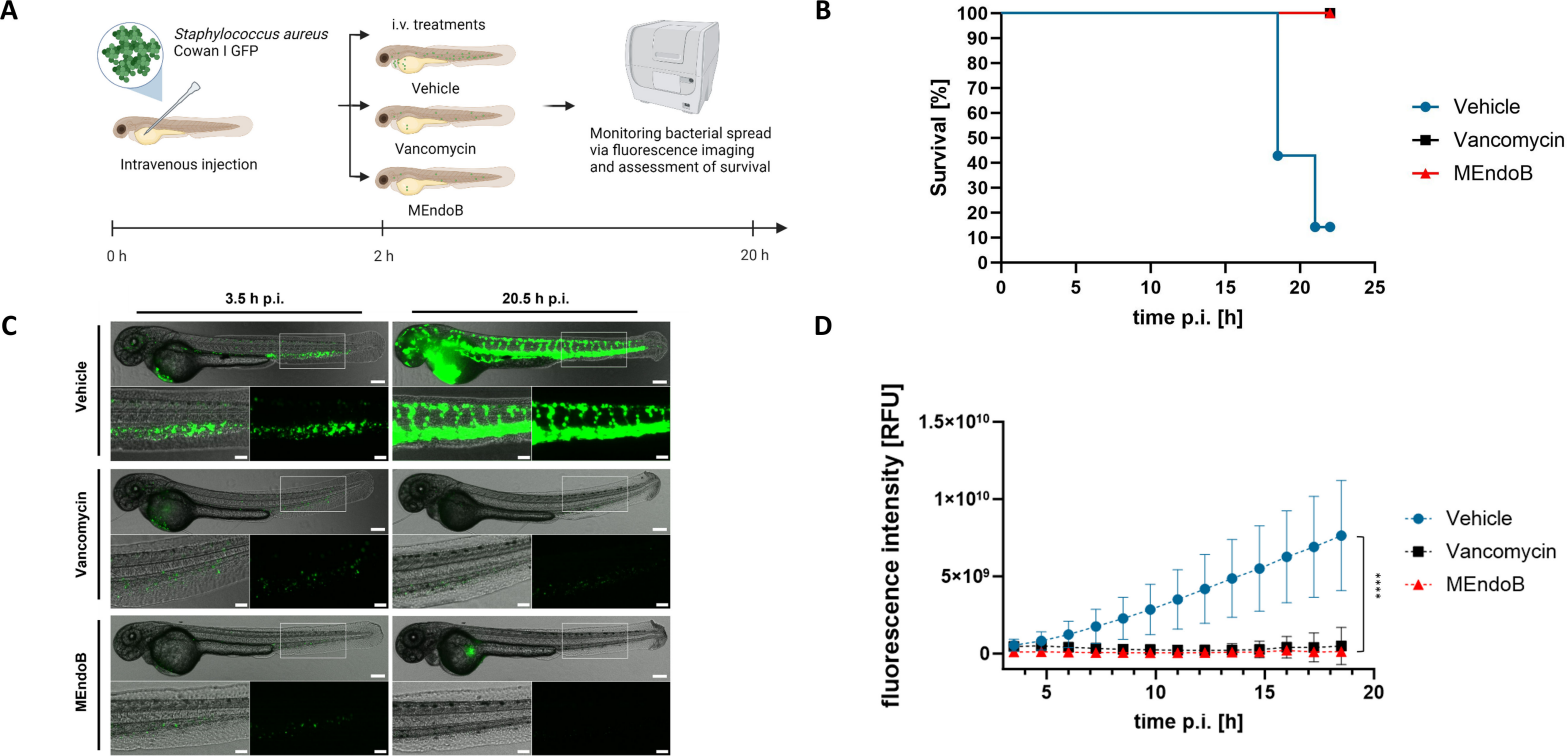
MEndoB rescues zebrafish larvae infected with *S. aureus*. Overview of the experimental procedure (**A**). Forty-eight-hour-old zebrafish larvae were injected with *S. aureus* Cowan I pCN56_GFPmut2 (2.5 × 10^4^ CFUs/fish) and treated with vehicle (PBS), vancomycin, or MEndoB 2 h post-infection. Survival of treated zebrafish larvae was monitored (*n* = 7) (**B**). Real-time GFP fluorescence images were overlayed with phase contrast microscopy images (**C**) and their intensity was quantified over time (**D**). Scale bar = 500 mm, scale bar of insets = 200 mm. Mean ± SD is shown (*n* = 7). ****P ≤ 0.0001, repeated measure two-way ANOVA.

Having demonstrated MEndoB activity *in vitro* in human and various animal sera and *ex vivo* in human whole blood samples, we set out to assess the enzyme’s potential in different *in vivo* models of bacterial infection. In the first step, MEndoB efficacy was studied in zebrafish larvae infected with 2.5 × 10^4^ CFUs of a fluorescent *S. aureus* strain (Cowan I pCN56_GFPmut2), 48 h post-fertilization (Fig. 4A; Videos S1 and S2). MEndoB (4 ng/fish), vancomycin (4 ng/fish, positive control), or PBS (vehicle, negative control) were micro-injected into zebrafish larvae via the duct of Cuvier, 2 h post-bacterial infection. *S. aureus* infections were lethal after 21 h in six out of seven zebrafish in the vehicle group, whereas all MEndoB-treated zebrafish survived, demonstrating MEndoB *in vivo* efficacy (Fig. 4B). Real-time microscopic imaging of GFP fluorescence over time showed a significant reduction in MEndoB-treated zebrafish larvae compared to PBS-treated ones (Fig. 4C and D). The vancomycin-treated, positive control group showed very similar survival and fluorescence microscopy results to the MEndoB group (Fig. 4B through D).

### MEndoB rescues mice from a lethal systemic *S. aureus* infection

**Fig 5:**
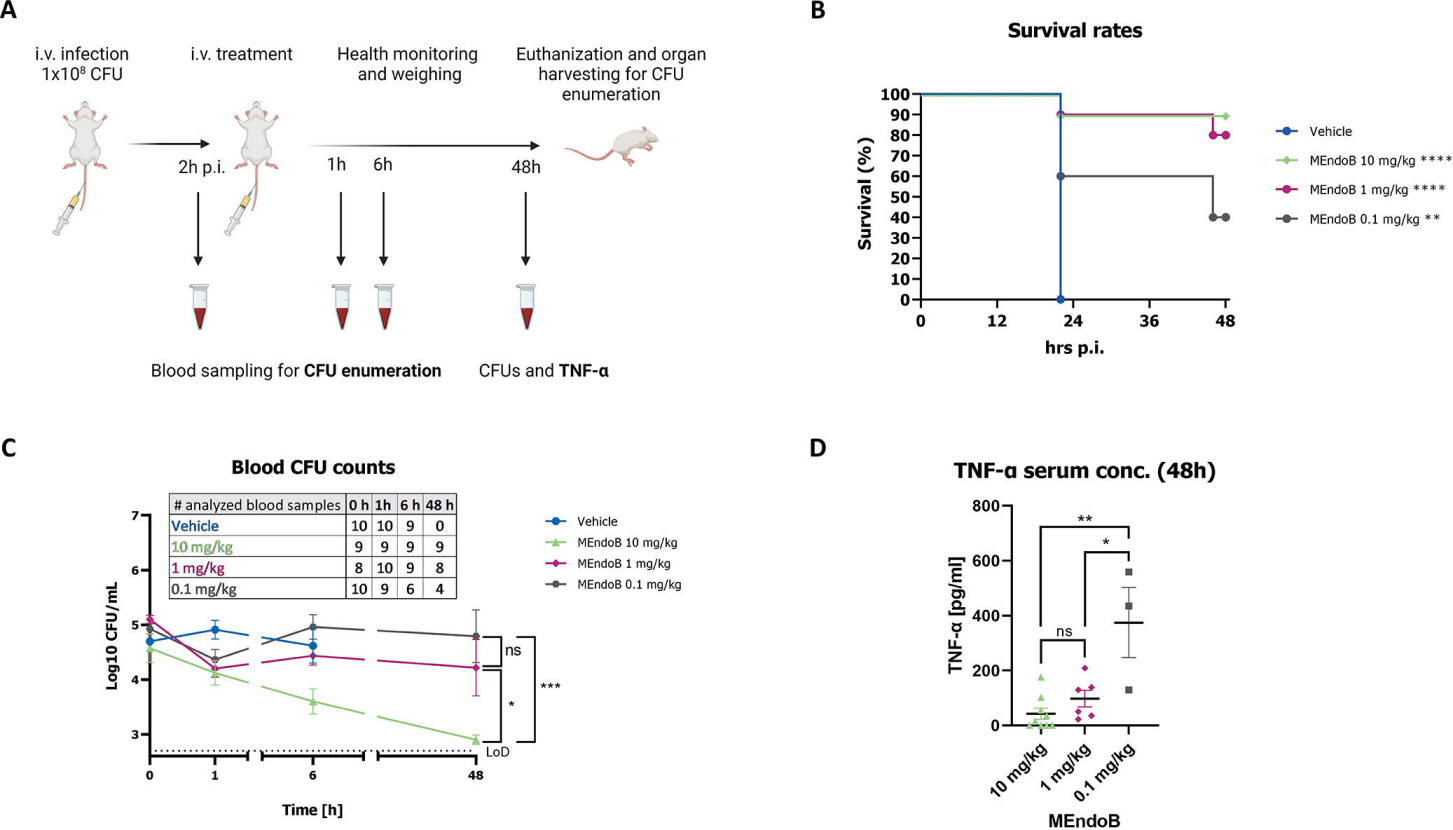
Treatment with MEndoB results in dose-dependent survival of up to 90% in a lethal mouse bacteremia model. Schematic representation of the experimental procedure (A). Survival rates of mice infected with *S. aureus* USA300 LAC strain treated with vehicle (PBS) or MEndoB at 0.1 mg/kg (gray), 1.0 mg/kg (pink), and 10.0 mg/kg (green) were monitored over 48 h post-treatment (*n* = 10 per group) (B). CFUs in the blood of treated animals were analyzed after 0, 1, 6, and 48 h (C). The limit of detection was 2.7 Log10 CFUs/mL (dotted line). The number of analyzed blood samples is shown in the table; only non-deceased animals with clinical scores permitting blood sampling were included in the analysis (no vehicle group beyond 6 h time point). TNF-α serum concentration in the blood of the mice infected with *S. aureus* USA300 LAC strain was analyzed 48 h post-treatment (D). For all graphs, error bars indicate the standard error of the mean. Statistical analysis performed for survival rates was a log-rank (Mantel-Cox) test (vehicle vs treatment groups) and an unpaired *t*-test at 48 h for blood CFU counts and TNF-α concentrations (****P < 0.0001, ***P ≤ 0.001, **P ≤ 0.01, *P ≤ 0.05, and ns > 0.05).

To further evaluate the therapeutic potential of MEndoB *in vivo*, we used a lethal systemic mouse bacteremia model (48). The effect of MEndoB on survival, *S. aureus* CFU counts, and levels of the cytokine tumor necrosis factor alpha (TNF-α), a marker for infection and inflammation (49), were determined for three different single doses over 48 h (Fig. 5A).

Infected animals were treated with MEndoB 2 h post-infection and continuously monitored for 48 h. While all control mice had to be sacrificed or deceased after 24 h, a dose-dependent survival (48 h) of up to 90%, 80%, and 40% was observed for the three doses of MEndoB ranging from 10, 1, and 0.1 mg/kg, respectively (Fig. 5B). CFU counts in the blood decreased after 1 h for all doses and the highest dose exhibited a significant reduction after 6 h, which was sustained until the end of the experiment (Fig. 5C). No signs of a negative impact of the PGHs on the infected animals were observed. MEndoB showed a dose-dependent effect on cytokine TNF-α concentration measured at 48 h post-infection, with concentrations in the 10 mg/kg and 1 mg/kg groups significantly lower than those in the 0.1 mg/kg group. The TNF-α concentration in the vehicle group could not be determined due to the death of all animals in that group at 24 h (Fig. 5D). In summary, the observed survival rates, CFU levels, and TNF-α concentration matched, suggesting dose-dependent killing of *S. aureus in vivo*, resulting in reduced inflammation and increased survival.

## DISCUSSION

MEndoB was selected by a systematic screening procedure from a chimeric PGH library tailored toward the treatment of systemic staphylococcal infections. Its characterization revealed superior activity compared to other published staphylolytic PGHs, a low MIC range against six different staphylococcal species including clinical isolates, and synergy with conventionally used antibiotics. The high activity of MEndoB in human and animal sera of seven different animals, and in human whole blood, makes it a promising candidate for further assessment. Its high therapeutic potential was corroborated in lethal *S. aureus* zebrafish larvae and mouse infection models. The latter showed a dose-dependent decrease in CFU numbers and TNF-α levels in the blood, resulting in survival rates of over 90%.

Approaches describing the screening of PGH libraries for enzymes with desired properties have previously been presented and reviewed (32, 41, 43, 44, 50) and learnings from the previous work laid the foundation for the study presented here. In particular, Sobieraj et al. (44) identified chimeric PGHs displaying high activity in human serum and whole blood, including a construct featuring a combination of a CHAP and an M23 domain within the same protein. Building on this finding, we created a library of chimeric PGHs, comprising CHAP and M23 domains from different origins. From this library, we selected and characterized the second-generation PGH, MEndoB, for its high activity *in vitro*, *ex vivo,* and *in vivo*. MEndoB demonstrated superior activity compared to the non-chimeric and non-engineered first-generation endolysins PlySs2 and SAL-1 (51). PlySs2 and SAL-1 have previously been comprehensively characterized and were studied in clinical trials (52–54). In addition to the higher activity observed in a head-to-head comparison using qKAs (Fig. 1D and E), the MIC values for MEndoB (0.008–0.5 µg/mL) were lower than those reported in the literature for PlySs2 (0.25–1 µg/mL) and SAL-1 (0.22–3.24 µg/mL) (46, 55) (Table 1). This supports the hypothesis that MEndoB may also be more active in advanced models and illustrates the value of a thorough lead candidate selection.

Preclinical *in vivo* models are frequently used to predict treatment efficacy for later stages of development. The zebrafish larvae infection model used in this study presents a novel way to study bacterial infection and PGH treatment efficacy in an early *in vivo* setting, which already features an innate immune system (56, 57). We demonstrated the survival of MEndoB-treated zebrafish larvae and used the possibility to visualize bacterial infection and PGH treatment *in vivo* via real-time fluorescence microscopy, which this model offers (Fig. 4; Videos S1 and S2). In this manner, the rapid and effective action of MEndoB against *S. aureus*, which was comparable to the positive control, vancomycin, was visualized. While vancomycin worked effectively against the *S. aureus* strain used in this study, the prevalence of vancomycin-resistant strains in treatment settings is rising and has more than doubled from the decade before 2010 to the one after (58). Thus, the development of new solutions against resistant bacterial strains is crucial. PGHs are known to work independently of the strain’s resistance patterns (59) and we here confirmed that MEndoB works against staphylococci, independent of their resistance against methicillin (Table 1; Fig. 3). This is due to the active killing mechanism of PGHs, which target highly conserved PG structures of staphylococci and have the additional advantage of a rapid onset of killing (Fig. 3). In addition, MEndoB combines the high activity of two EADs in one chimeric protein, which has previously been shown to reduce the probability of resistance development (42). Thus, MEndoB offers advantages over existing antibiotics and could be a solution against staphylococci, including resistant strains. To further explore the potential of MEndoB and additionally assess the transferability of the zebrafish larvae infection model, we next tested MEndoB in a lethal murine *S. aureus* infection model. MEndoB low (0.1 mg/kg), intermediate (1.0 mg/kg), and high (10.0 mg/kg) doses led to survival rates of 40%, 80%, and 90%, respectively. Thus, both *in vivo* models showed a survival response, and more elaborate transferability studies could support the reduction of animal testing in bigger model organisms in the future (60). In similar mouse studies with PlySs2, 2.5 and 5.0 mg/kg were necessary to achieve 20% and 70% survival after 24 h, respectively (61), suggesting that the higher *in vitro* activity of MEndoB also translated to more advanced *in vivo* models.

*S. aureus* infections are the leading cause of antimicrobial resistance (AMR)-related deaths in high-income countries and the second most common cause of AMR-related deaths worldwide (5, 16). While hygiene and antibiotic stewardship are important pillars to delay the spread of resistance, there is an urgent unmet need for truly novel antimicrobial treatments with new modes of action to fight bacteria that are already multi-resistant and affect patients today. To facilitate the selection of appropriate pre-clinical models in the future, the activity range of MEndoB across seven different animal sera was determined in qKAs (Fig. 2A and B). In contrast to findings for other PGHs, such as PlySs2, robust activity of MEndoB across all tested animal sera was observed (47). While a more detailed examination will be necessary, this could lead to a more predictable dose-effect relationship in animal studies with MEndoB, where previous studies used varying PGH doses, depending on the animal model employed (61–63). The observed synergy and additive effects between MEndoB and conventionally used antibiotics could serve as the basis for such further *in vivo* investigations and should therefore be included therein (Table 1). In this study, we demonstrated activity at elevated temperatures that may be found in patients with systemic infections (Fig. 2C) and good in-use stability (Fig. 2D), which are some important requirements of new protein-based antimicrobials and emphasize the potential of MEndoB.

MEndoB was engineered to target different staphylococcal species as demonstrated in qKAs, MICs, and killing assays in human whole blood (Fig. 1C and E; Fig. 2B and D, Fig. 3C and D; Table 1). In addition to systemic staphylococcal infections, the high specificity of endolysin-derived PGHs, such as MEndoB, could be of advantage when treating skin-related diseases (64, 65) or in the prevention of infections of indwelling devices, implants, or joints where staphylococci often form biofilms after surgery (66). Other PGHs have shown activity against biofilms, and the potential of MEndoB against biofilms should be confirmed in future studies (31). Thus, the possible applications of MEndoB extend beyond systemic staphylococcal infections.

In summary, the presented chimeric, second-generation PGH, MEndoB, unites fundamental characteristics required of a new antimicrobial, such as good in-use stability, rapid and effective killing in human blood and in two *in vivo* SBI models. More extensive preclinical characterizations of MEndoB will be necessary in the future to confirm its high potential and ultimately move toward clinical assessment.

## MATERIALS AND METHODS

### Bacterial strains and growth conditions

Bacterial strains that were used in this study are listed in Table S1. Staphylococcal strains were grown by shaking at 180 rpm in tryptic soy broth (TSB, pH 7.3) at 37°C or as specified in the specific assay section. *Escherichia coli* strains were cultured in Luria-Bertani (LB) medium (10 g/L tryptone, 5 g/L yeast extract, and 10 g/L NaCl, pH 7.4) supplemented with tetracycline (20 µg/mL) and kanamycin (50 µg/mL) for pET200 expression plasmid selection. *E. coli* and ClearColi expression was done in LB-PE optimized for protein expression (15 g/L tryptone, 8 g/L yeast extract, and 5 g/L NaCl, pH 7.8), LB-PE optimized for ClearColi protein expression (15 g/L tryptone, 8 g/L yeast extract, and 10 g/L NaCl, pH 7.8), respectively, or JS10 medium [30 g/L yeast extract, 11 mM citric acid, 3 mM MgSO_4_, 100 mM ammonium phosphate dibasic, 1% (vol/vol) glycerol, 0.1% (vol/vol) trace elements A, and 0.1% (vol/vol) trace elements B, pH 7.0] (43, 67).

### Molecular cloning

The screening library contained EADs and CBDs of highly active PGHs and was assembled using standard molecular biology methods. CHAP domains of the endolysins of phage GH15 (68), Twort (36), K (69), and SEP (70), as well as the bacteriocins LST (71) and ALE1 (72), were included. For the CBDs, a selection of the domains from the endolysin of phage 2638A, as well as those of the bacteriocins LST and ALE1, were integrated. MEndoB contains the CHAP domain of the endolysin of the staphylococcal phage Twort (GenBank: AAX92311.1) and the M23 and SH3b domain of the bacteriocin lysostaphin (GenBank: AAA26655.1). A domain linker (SVKKKDTKKKPKPSNRDGINKDK) from the staphylococcal phage Twort (GenBank: AAX92311.1) was used to connect the N-terminal CHAPTw domain to the central M23LST domain, and the natural linker from lysostaphin (KAGGTVTPTPNTG) (GenBank: AAA26655.1) was used to connect the central M23LST domain to the C-terminal SH3bLST domain. In addition, a His-Tag version of MEndoB was engineered (MEndoB_H) by adding a C-terminal 6× His-Tag. The protein PlySs2_H contains the CHAP and SH3b domain of *Streptococcus suis* strain 89/1591 (GenBank: ZP_03625529) (45) and a C-terminal 6× His-Tag. The protein SAL-1 consists of a CHAP, amidase-2, and SH3b domain of the bacteriophage SAP-1 (46) and a C-terminal 6× His-Tag. All DNA fragments were purchased from GeneArt GmbH and cloned into the pET200 vector by using the Gibson assembly technique (73). The plasmids were transformed into *E. coli* BL21-Gold(DE3) or ClearColi and their construct identity was confirmed by commercial Sanger sequencing.

### Protein expression and purification

Expression of recombinant proteins in *E. coli* and ClearColi was carried out as previously described (43). In brief, bacterial cultures were grown shaking (180 rpm) in LB-PE at 37°C and supplemented with suitable antibiotics for plasmid selection until an OD_600_ of 1.0 was reached. Following cooling on ice, 0.5 M isopropyl-β-D-thiogalactopyranoside was added and incubation under agitation was continued for 18 h at 20°C. Cells were centrifuged, washed in wash buffer for cation exchange chromatography (CIEX) (50 mM Na_2_HPO_4_, 50 mM NaCl, and 20% [vol/vol] glycerol, pH 7.4) and frozen at −80°C. Pellets were resuspended in wash buffer and cells were disrupted by one passage through a high-pressure homogenizer at 17.5 KPSI (Cell disruptor CF1, I&L Biosystems). Proteins were purified by CIEX as previously described (43), using a HiTrap Sepharose fast-flow (SP-FF) column on a fast protein liquid chromatography device (Äkta Pure 25 L, GE Healthcare) and eluted with a 1%/ min gradient of CIEX elution buffer (50 mM Na_2_HPO_4_, 1 M NaCl, and 20% [vol/vol] glycerol, pH 7.4). Proteins with His-Tag were purified by immobilized metal affinity chromatography (IMAC) using a HisTrap FF column with IMAC wash buffer (50 mM Na_2_HPO_4_, 300 mM NaCl, and 10 mM imidazole, 30% [vol/vol] glycerol, pH 8.0) and a step elution of 6% of IMAC elution buffer (50 mM Na_2_HPO_4_, 300 mM NaCl, 250 mM imidazole, and 30% [vol/vol] glycerol, pH 8.0) for 4 CV followed by 100% IMAC elution buffer for 8 CV. Depending on the application, eluted proteins were further dialyzed into appropriate buffers, filter sterilized (0.2 µm), and protein concentration was measured with a spectrophotometer (NanoDrop One^C^, Thermo Fisher Scientific). Protein identity and purity were evaluated by SDS-PAGE. For *in vivo* experiments, the purification procedure described above was modified to yield endotoxin-free preparations, as previously described (74). Three-dimensional protein structures were predicted using ColabFold (75) based on DeepMind’s AlphaFold (76) and visualized via PyMOL Molecular Graphics System (Graphics System, Version 2.5.4 Schrödinger, LLC).

### PGH functional assays

#### Turbidity reduction assay

Turbidity reduction assays were performed as previously described (44). Frozen *S. aureus* or *S. epidermidis* cells were thawed and diluted in PBS-T (7 mM Na_2_HPO_4_, 3 mM NaH_2_PO_4_, 130 mM NaCl, and 0.01% Tween 20, pH 7.4) or human serum and aliquots of the suspension were mixed in a 96-well plate with PGH dilutions in the same buffer/serum, with final concentrations ranging from 5 to 40 nM for *S. aureus* and 10–80 nM for *S. epidermidis*, so that the initial OD_600_ of the suspensions was 1.0. Buffer/serum without PGH served as a negative control. The optical density in each well was monitored for 25 min (151 cycles) at 10-s intervals using a plate reader (MultiSkan Sky, Thermo Fisher Scientific) by shaking at 30°C. The resulting lysis curves were normalized and corrected for the no-PGH control. The integrals of these control-corrected lysis curves were calculated over 25 min for each tested concentration. To compare the activity of PGHs, the mean of the integrals from the tested concentrations was determined and inversed. The standard error of the mean was calculated from two, three, or four biological replicates depending on the PGH selection process, strain, and buffer.

#### Quantitative killing assay in human and animal sera

MEndoB was tested against *S. aureus* ATCC 12600 and *S. epidermidis* ATCC 35984. The final inoculum was aimed at 10^6^–10^7^ CFUs/mL. Precultures were prepared in the TSB medium, followed by overnight (O/N) incubation at the conditions described above. Suitable aliquots of sera (human, pig, cow, horse, dog, mouse, rat, and rabbit) were thawed at 30°C, filtered (0.45 µm), and stored on ice. O/N cultures were diluted in TSB and incubated at standard conditions until reaching the exponential growth phase (OD_600_ of 0.5–0.6). Bacterial cultures were adjusted with TSB to an OD_600_ of 0.5 after cooling to stop further growth and diluted 1:10 in serum. A twofold serial dilution of MEndoB in serum was prepared in a 96-well F-bottom plate (Bioswisstec AG). The bacterial suspension was added to the wells, including a growth control, and the plate was incubated at 37°C and 180 rpm for 30 min. After incubation, 10× stopping buffer (386.8 mM trisodium citrate and 647.2 mM citric acid) was subsequently added and mixed to stop enzymatic activity. Each well was serially diluted in 1× stopping buffer, spot-plated on LB agar plates, and incubated O/N at 37°C. Viable cell count and CFUs/mL log reduction across the tested final MEndoB concentration range were visualized.

#### Stability-qKA

To assess a potential loss in activity over time, MEndoB was prediluted in human serum in low-protein binding tubes (Protein LoBind, Eppendorf) and incubated at 37°C for 4 h before activity testing. A qKA was then performed at *T* = 4 h for 30 min at 37°C and 180 rpm as described above and compared to MEndoB that had not been pre-treated.

#### MIC determination by broth microdilution

The CLSI-approved MIC method using cation-adjusted Mueller-Hinton broth supplemented with 25% horse serum and 0.5 mM DL-dithiothreitol (CAMHB-HSD) had previously been developed for testing of a PGH against *S. aureus*, was employed to determine MICs (55). Briefly, CAMBH-HSD was prepared freshly and sterile filtered. Staphylococcal strains were inoculated to reach a final concentration of around 1–7 × 10^5^ CFUs/mL and were mixed with enzyme dilutions. For all assays, 96-well U-Bottom plates (Bioswisstec AG) were used. Assay plates were incubated for 18 h at 35°C in ambient air and MICs were determined via OD_600_ measurement of resuspended wells (Multiskan Sky, Thermo Fisher Scientific). The MIC was defined as the lowest concentration (in μg/mL) at which the OD_600_ was still below a threshold of 0.01 (no growth observed). For each strain, at least three biological replicates were performed. MICs of methicillin-resistant and susceptible strains were compared using an unpaired *t*-test.

#### Checkerboard assay

Checkerboard assays were performed as previously described with minor modifications (77). Briefly, *S. aureus* ATCC 12600 was inoculated in CAMHB-HSD or CAMHB-HSD supplemented with CaCl_2_ (50 mg/mL) for daptomycin (DAP) testing to a final concentration of around 10^5^ CFUs/mL. For other antibiotics (flucloxacillin, oxacillin, levofloxacin, vancomycin, cefoxitin, DAP, and trimethoprim/sulfamethoxazole) and MEndoB, solutions were prepared at desired concentrations in testing medium (CAMHB-HSD or CAMHB-HSD supplemented with 50 mg/mL CaCl_2_ for DAP testing) in sterile U-bottom 96-well plates each with dilutions following rows or columns, respectively. To prepare the checkerboard plates, the first compounds’ dilutions were transferred to a new, sterile U-bottom 96-well plate while retaining the order of the wells. Additionally, the second compounds’ dilutions were transferred and mixed with the first compounds’ dilutions while retaining the order of wells. Plates were incubated overnight at 35°C for 18 h. After incubation, bacteria were resuspended, and OD_600_ was measured to determine wells with bacterial growth. Based on the growth inhibition pattern, the fractional inhibitory concentration index was calculated by using the following equation: A/MIC_A_ + B/MIC_B_ = FICA + FICB = FIC Index, where A and B are the MICs of each antibiotic in combination (in a single well), and MIC_A_ and MIC_B_ are the MICs of each drug individually. The FICI value was then used to categorize the interaction of the two antimicrobial substances tested. Synergy: FIC index ≤ 0.5; additive or indifference > 0.5–4; antagonism > 4 (55, 78).

### *Ex vivo* activity of MEndoB in fresh human whole blood

Blood samples from healthy volunteers (21–41 years of age, male and female) were collected at the Department of Infectious Diseases and Hospital Epidemiology at the University Hospital Zurich. *Ex vivo* activity testing in fresh human whole blood was performed by modifying a previously described procedure (79). Briefly, freshly drawn whole blood was aliquoted into low-protein binding tubes (Protein LoBind, Eppendorf) and spiked with *S. aureus* ATCC 12600 (MSSA) or USA300 JE2 (MRSA) or *S. epidermidis* 1457 (MSSE) or ATCC 35984 (MRSE) at a concentration of 10^4^ CFUs/mL. MEndoB was added at a concentration of 10, 50, and 100 nM for *S. aureus* and 40, 200, and 400 nM for *S. epidermidis*. Samples were incubated at 37°C with constant shaking. CFUs were determined by plating and overnight incubation at 37°C of serial dilutions of blood in water at defined time points on TSA plates. Differences in the killing of methicillin-resistant and methicillin-susceptible strains were compared using unpaired *t*-tests for matching concentrations and time points.

### Zebrafish model of bacterial infection

Zebrafish larvae infection models were essentially performed as previously described (60, 80). Fertilized zebrafish (*Danio rerio*) eggs were obtained from adult wild-type (AB/TU) fish, washed and maintained at 28°C in fish buffer (5 mM NaCl, 0.25 mM KCl, 0.5 mM MgSO_4_, 0.15 mM KH_2_PO_4_, 0.05 mM Na_2_HPO_4_, 0.5 mM CaCl_2_, and 0.71 mM NaHCO_3_) containing 0.003% (wt/vol) 1-phenyl 2-thiourea to prevent pigmentation. Forty-eight hours post-fertilization, zebrafish larvae were dechorionated and subsequently anesthetized using 0.01% (wt/vol) buffered tricaine. Fish larvae were then embedded in 0.2% (wt/vol) buffered agarose supplemented with 0.01% (wt/vol) tricaine for immobilization. *S. aureus* (Cowan I pCN56_GFPmut2) overnight cultures (in LB medium supplemented with 10 µg/mL erythromycin [AppliChem Panreac]) were diluted 1/100 and re-incubated for 5 h. In their exponential growth phase, bacteria were washed, diluted in PBS to a concentration of 2.5 × 10^10^ CFUs/mL, and micro-injected into zebrafish larvae into the duct of Cuvier in a total volume of 1 nl. Injected CFU numbers were confirmed by dilution plating on LB agar. After 2 h, infected larvae were injected with PBS (vehicle), MEndoB (4 ng/fish), or vancomycin (4 ng/fish) and mounted for fluorescence microscopy imaging starting 3.5 h post-infection. Adult fishes were kept in accordance with Swiss animal-welfare regulations.

### Real-time monitoring of bacterial infections in zebrafish larvae by fluorescence imaging

Real-time epifluorescence imaging was performed using the Biotek Cytation 5 Cell Imaging Multi-Mode Reader (Agilent) set to 30°C. Images were acquired every 15 min using the system’s inverted microscope with a 4× objective (Olympus Plan Fluorite phase, NA, 0.13) in the GFP (ex. 469/35 and em. 525/39) or phase contrast channel for up to 21 h. Quantification of GFP fluorescence was performed using the Biotek Cytation software Gen5, and image processing was carried out with Fiji, ImageJ (81). A repeated measure two-way ANOVA test was performed using GraphPad Prism for statistical analysis.

### Murine infection model

All animal experiments were conducted at Selvita, Croatia. Female and male BALB/c mice (10 weeks of age) (Charles River) were infected with 1 × 10^8^ CFUs of *S. aureus* USA300 LAC (exponential growth phase) by injection into the tail vein. At 2 h post-infection, mice were treated with 100 µL of MEndoB in three concentrations (10, 1, and 0.1 mg/kg) administered via the tail vein. Control mice received 100 µL of sterile Dulbecco's PBS. Each treatment group consisted of five male and five female mice. Blood was drawn from the tail vein 1, 6, and 48 h post-treatment if clinical conditions allowed. Mice were monitored twice daily and clinically scored. Mice were sacrificed after reaching a predefined humane endpoint. No blood sampling could be performed for animals that succumbed to the infection or showed critical clinical symptoms. Collected blood aliquots were plated on Mueller-Hinton agar to evaluate the load of *S. aureus* in the bloodstream. Terminal blood sampling was performed via jugular vein bleed 48 h post-infection from all surviving animals at the end of the study. Mice were sacrificed by terminal exsanguination under ketamine + xylazine anesthesia. Sera were prepared from coagulated blood samples by centrifugation at 3,500 rpm for 15 min at 4°C, separated, and stored at −80°C. TNF-α concentration in serum was determined via ELISA.

## AFTER EPUB

*[This article was published on 26 January 2024 with a missing citation in Table S1. The table was corrected in the current version, posted on 2 February 2024.]*

## SUPPLEMENTAL MATERIAL

Supplemental Movie and Table Legends mbio.02540-23-s0001.docx Legends for Videos S1 and S2 and Table S1.

Supplemental figures mbio.02540-23-s0002.pdf Fig. S1-S4.

Table S1 mbio.02540-23-s0003.xlsx Bacterial strains used in this study.

Video S1 mbio.02540-23-s0004.mp4 Infected zebrafish larvae treated with PBS.

Video S2 mbio.02540-23-s0005.mp4 Infected zebrafish larvae treated with MendoB.

ASM does not own the copyrights to Supplemental Material that may be linked to, or accessed through, an article. The authors have granted ASM a non-exclusive, world-wide license to publish the Supplemental Material files. Please contact the corresponding author directly for reuse.
