# Supplementary material for: MEndoB, a chimeric lysin featuring a novel domain architecture and superior activity for the treatment of staphylococcal infections
Source: mBio. 2024 Jan 26;15(2):e02540-23. doi: 10.1128/mbio.02540-23 (PMC10865858; doi:10.1128/mbio.02540-23)
Supplement: Supplemental Movie and Table Legends — Legends for Videos S1 and S2 and Table S1. [file mbio.02540-23-s0001.docx]

**Legends for supplementary movies and tables**

***Supplementary Movie 1| Infected zebrafish larvae treated with PBS***

*Zebrafish larvae infected with S. aureus (Cowan I pCN56_GFPmut2, 2 x 10^4^ CFU/ fish) and, after 2 h, treated with PBS (vehicle control). Overlays of phase contrast and EGFP images (left) and fluorescence images only (right) are shown. Time refers to hours post PBS treatment. Scale = 500 mm.*

***Supplementary Movie 2| Infected zebrafish larvae treated with MEndoB***

*Zebrafish larvae infected with S. aureus (Cowan I pCN56_GFPmut2, 2 x 10^4^ CFU/ fish) and, after 2 h, treated with MendoB. Overlays of phase contrast and EGFP images (left) and fluorescence images only (right) are shown. Time refers to hours post MEndoB application. Scale = 500 mm.*

***Supplementary Table 1| Bacterial strains used in this study.*** Methicillin resistance status, strain origin and clinical isolate ID are shown for clinical isolates and reference strains. Clinical isolates were obtained from Streek Lab, Regional Public Health Laboratory Kennemerland, Haarlem (NL).
