# Supplementary material for: MEndoB, a chimeric lysin featuring a novel domain architecture and superior activity for the treatment of staphylococcal infections
Source: mBio. 2024 Jan 26;15(2):e02540-23. doi: 10.1128/mbio.02540-23 (PMC10865858; doi:10.1128/mbio.02540-23)
Supplement: Supplemental figures — Fig. S1-S4. [file mbio.02540-23-s0002.pdf]

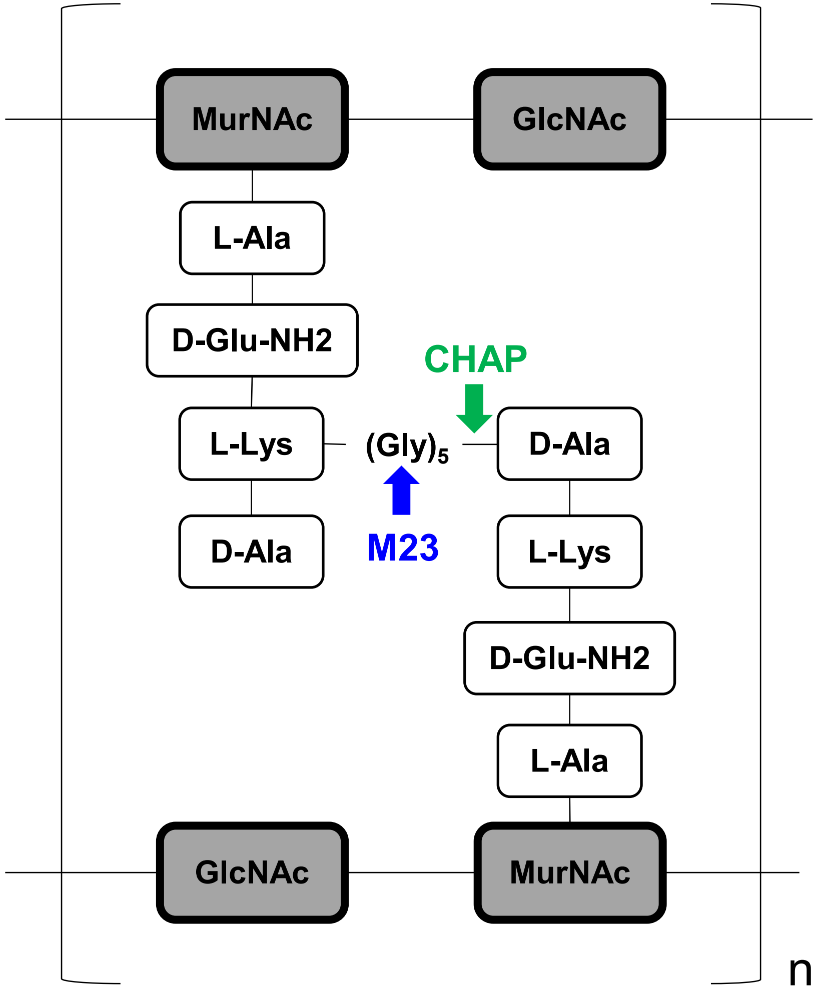

Supplementary Figure 1| MEndoB cut sites in peptidoglycan.
Arrows indicate the cleavage sites in the staphylococcal peptidoglycan which are targeted by the two enzymatically active domains (EADs) of MEndoB.

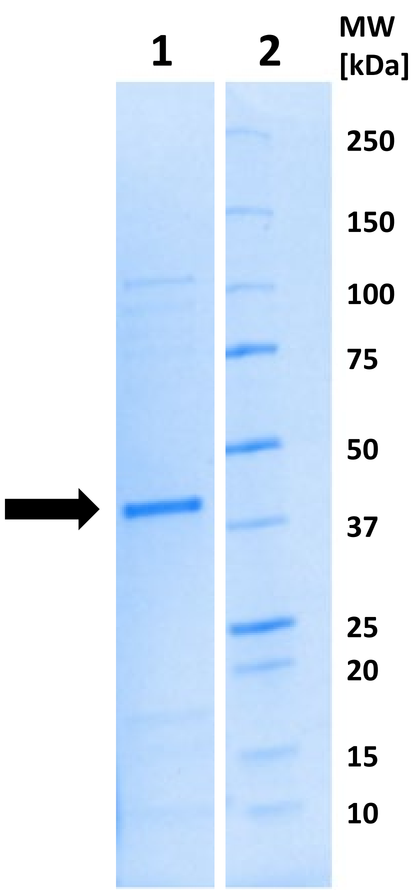

Supplementary Figure 2| SDS-PAGE gel of CIEX-purified MEndoB. Coomassie stained sodium dodecyl sulfate polyacrylamide gel electrophoresis (SDS-PAGE) gel showing MEndoB at expected size (black arrow). MEndoB (lane 1) and molecular weight marker (Precision Plus Protein Unstained Standard, BioRad; lane 2).

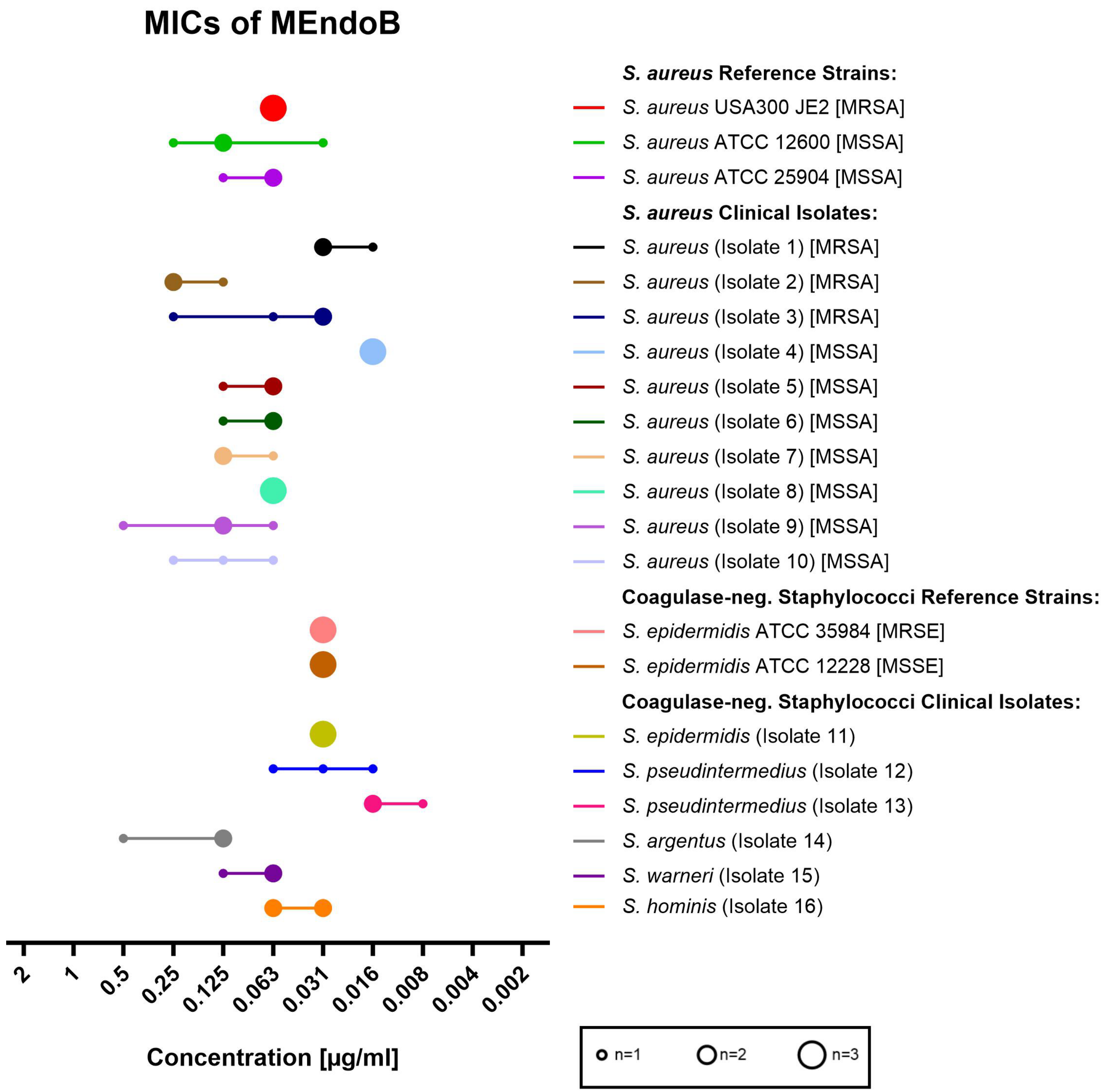

Supplementary Figure 3| Minimum Inhibitory Concentration (MIC) assessment demonstrates successful inhibition of various staphylococcal strains including methicillin-sensitive and -resistant clinical isolates.

MIC results for MEndoB against clinical isolates and reference staphylococcal strains. MICs against *S. aureus* and coagulase-negative staphylococcal species such as *S. epidermidis* were assessed in CAMHB-HSD following the CLSI guidelines. At least three biological replicates were performed. Evaluated and weighted (number of biological replicates) MIC values of MEndoB against each strain are shown in the graph as dots. Size of dots corresponds to the number of biological replicates (n) showing a certain MIC value.

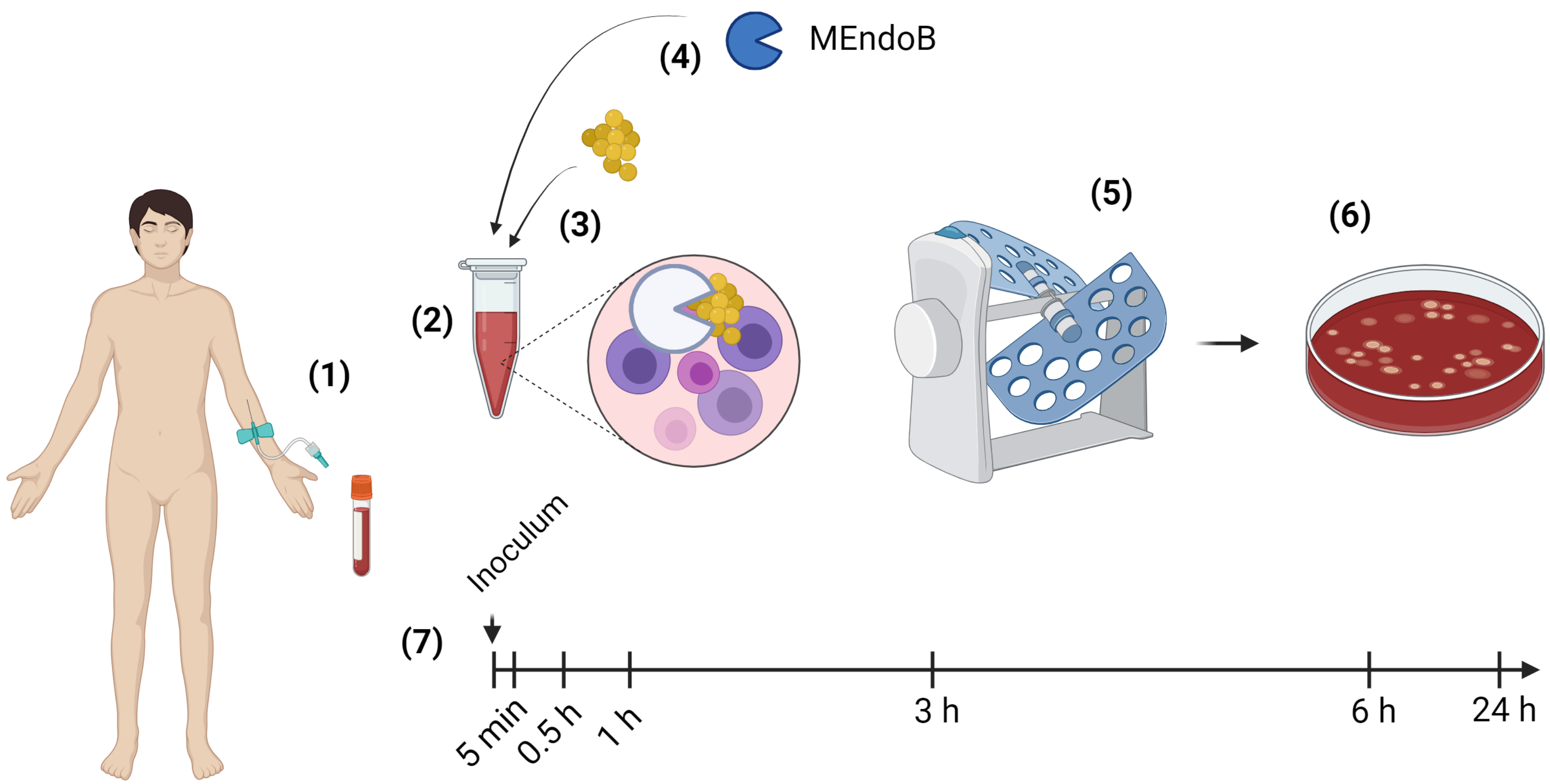

Supplementary Figure 4| Schematic procedure of testing MEndoB in whole blood (ex vivo).
Human whole blood was sampled from healthy donors (1). Fresh heparinized human blood was immediately aliquoted into protein low binding microtubes (2). *S. aureus* (strain ATCC 12600 or USA300 JE2) or *S. epidermidis* (strain 1457 or ATCC 35984) were inoculated at a final concentration of 10^4 CFUs/ml (3). MEndoB was added to the blood at different concentrations (10nM, 50nM and 100nM or 40nM, 200nM and 400nM for *S. aureus* and *S. epidermidis*, respectively) (4). The samples were incubated at 37 °C with overhead rotation (5). Counts of viable bacteria were determined by serial dilutions of the infected blood samples in dH2O on Columbia sheep blood agar plates (6). Timeline shows different sampling time points (7). Figure was made with biorender.com.
